# Sculpting the maturation, softening and ethylene pathway: The influences of microRNAs on tomato fruits

**DOI:** 10.1186/1471-2164-13-7

**Published:** 2012-01-09

**Authors:** Jinhua Zuo, Benzhong Zhu, Daqi Fu, Yi Zhu, Yuanzheng Ma, Lihong Chi, Zheng Ju, Yunxiang Wang, Baiqiang Zhai, Yunbo Luo

**Affiliations:** 1 Laboratory of Postharvest Molecular Biology of Fruits and vegetables, Department of Food Biotechnology, College of Food Science and Nutritional Engineering, China Agricultural University, Beijing, 100083, China

## Abstract

**Background:**

MicroRNAs (miRNAs), a ubiquitous class of short RNAs, play vital roles in physiological and biochemical processes in plants by mediating gene silencing at post-transcriptional (PTGS) level. Tomato is a model system to study molecular basis of fleshy fruit ripening and senescence, ethylene biosynthesis and signal transduction owing to its genetic and molecular tractability. To study the functions of miRNAs in tomato fruit ripening and senescence, and their possible roles in ethylene response, the next generation sequencing method was employed to identify miRNAs in tomato fruit. Bioinformatics and molecular biology approaches were combined to profile the miRNAs expression patterns at three different fruit ripening stages and by exogenous ethylene treatment.

**Results:**

In addition to 7 novel miRNA families, 103 conserved miRNAs belonging to 24 families and 10 non-conserved miRNAs matching 9 families were identified in our libraries. The targets of many these miRNAs were predicted to be transcriptional factors. Other targets are known to play roles in the regulation of metabolic processes. Interestingly, some targets were predicted to be involved in fruit ripening and softening, such as Pectate Lyase, beta-galactosidase, while a few others were predicted to be involved in ethylene biosynthesis and signaling pathway, such as ACS, EIN2 and CTR1. The expression patterns of a number of such miRNAs at three ripening stages were confirmed by stem-loop RT-PCR, which showed a strong negative correlation with that of their targets. The regulation of exogenous ethylene on miRNAs expression profiles were analyzed simultaneously, and 3 down-regulated, 5 up-regulated miRNAs were found in this study.

**Conclusions:**

A combination of high throughput sequencing and molecular biology approaches was used to explore the involvement of miRNAs during fruit ripening. Several miRNAs showed differential expression profiles during fruit ripening, and a number of miRNAs were influenced by ethylene treatment. The results suggest the importance of miRNAs in fruit ripening and ethylene response.

## Background

Gene expression is regulated by transcriptional and post-transcriptional pathways, which are crucial for optimizing gene output and for coordinating cellular programs [1]. One of the recently discovered mechanisms in plants was short non-coding RNAs mediated gene silencing at post-transcriptional (PTGS) level [2]. Short RNAs (sRNAs) are diverse and can be categorized into two major classes: short interfering RNAs (siRNAs) and microRNAs (miRNAs) [3,4]. SiRNAs, processed from perfectly double-stranded RNA (dsRNA), posttranscriptionally silence transposons, viruses, and transgenes are important for DNA methylation [5-7]. MicroRNAs (miRNAs), a near ubiquitous class of short RNAs in plants, are orchestrated by DCL-like family from single-stranded RNA precursors that possess imperfect stem-loop foldback structures [8,9]. Base pairing is used by mature miRNAs to guide RISCs to specific mRNAs bearing fully or partly complementary sequences [10]. Repression of the target transcripts by miRNAs may occur through translational inhibition or slicing, with the two layers of regulation not necessarily coinciding spatially or temporally [11,12]. Based on the sheer abundance and diversity of plant miRNAs, it is likely that most, if not all, physiological and biochemical processes in plants involve at some point the action of one or more miRNAs [9,13,14].

Tomato is an emblematic system to study molecular basis of fleshy fruit ripening and senescence, ethylene biosynthesis and signal transduction owing to its genetic and molecular tractability [15-17]. Recently, involvement of sRNAs in tomato fruit has been received attention. The homology search and molecular biology methods were used for characterizing novel and conserved miRNAs at the outset [18-21]. As deep sequencing technology has emerged and been employed extensively to identify miRNAs in model plants such as Arabidopsis and rice because of its high throughputs and accuracy, which make explore miRNAs in large scale possible [22-25]. The 454 pyrosequencing sequencing platform was first used to sequence tomato sRNAs from young leaves and a young green fruits of *Microtom *[26]. Several conserved and non-conserved miRNAs were identified in this study, and miR156 was found to be involved in fruit ripening which raised the possibility that fruit ripening process may be under miRNA regulation [26]

Tomato fruit ripening and senescence are genetically regulated processes. Ripening of fleshy fruits involves evolution of ethylene, accumulation of pigments such as carotene and lycopene, development of aroma and flavor, softening of fruit tissues and increased susceptibility to pathogens [15,16,27]. To date, functional analysis has been carried out only for a few tomato miRNAs, most of which were validated to be involved in leaf and flower development [28-31], and a few of which were proved to be involved in the stress-response and host-pathogen interactions [32-35]. In order to study the functions of miRNAs in tomato fruit ripening and senescence, and their possible roles in ethylene pathway, the next generation sequencing method (Solexa platform) was employed to identify miRNAs in tomato fruit. Bioinformatics and molecular biology approaches were combined to profile the miRNAs expression patterns of fruits at three different ripening stages (mature green stage, breaker stage, red ripe stage). The influences of exogenous ethylene on the miRNAs expression levels were also explored.

## Results

### High-throughput sequencing of short RNAs of three fruit ripening stages

To explore the possible regulatory roles of miRNAs in fleshy fruit ripening process, we select fruits at three different ripening periods (mature green stage, the breaker stage and red ripening stage) of the *Solanum lycopersicum *(*Ailsa Craig*). The sRNA fraction was subject to deep sequencing on the Illumina (Solexa)1G platform which produced 4,333,963, 5,514,197 and 5,334,158 raw reads, respectively. After removal of the adaptor sequences and filtering by sequence properties, 2,388,170, 3,599,583 and 2,274,050 redundant sRNA reads were remained for further analysis, respectively (Table 1).

**Table 1:** Statistics of small RNA sequences from different tomato fruit ripening stages

Ripening stages	Reads types	Redundant	Non-redundant
	Raw reads	4,333,963	
	Low quality reads	1,562,982	
Mature green	Adaptor reads	382,811	
	High quality reads(≥ 18nt)	2,388,170	1,604,689
	Matching the genome	356, 350	141,075
	rRNA/tRNA/snRNA/snoRNA matches	65,593	11,177
	Raw reads	5,514,197	
	Low quality reads	1,728,593	
Breaker stage	Adaptor reads	186,021	
	High quality reads(≥ 18nt)	3,599,583	2,309,447
	Matching the genome	681,895	346,166
	rRNA/tRNA/snRNA/snoRNA matches	62,734	9,518
	Raw reads	5,334,158	
	Low quality reads	3,042,644	
Red Ripe stage	Adaptor reads	17,464	
	High quality reads(≥ 18nt)	2,274,050	1,447,762
	Matching the genome	437,520	220,188
	rRNA/tRNA/snRNA/snoRNA matches	29,865	4,750

**Figure 1:**
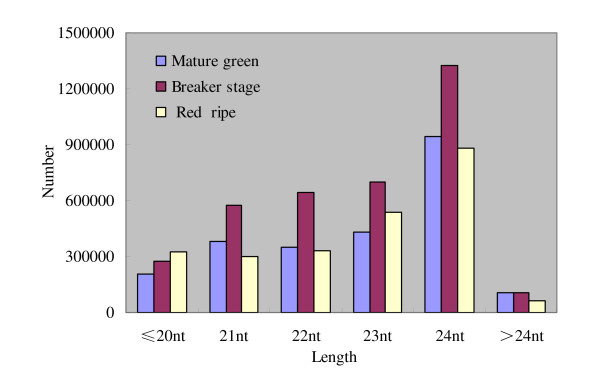
**Length comparison of small RNAs at three ripening stages of tomato fruit**. The length of the deep sequencing results were mainly between 21-24nt, the number of the 24nt sequences is obviously greater than the other sequence length.

**Figure 2:**
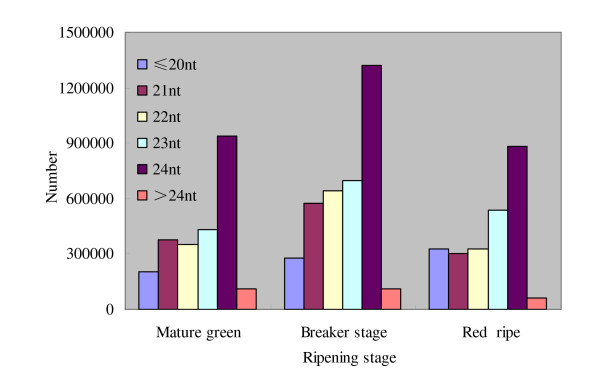
**Length distributions of small RNAs at three ripening stages of tomato fruit**. The overall sequences (21-24nt) in the breaker stage were more abundant than the other two ripening stages, the number of the 21-23nt sequences in each ripening stages showed no obvious difference, however, there was a sharp increase in the 24nt sequence number.

Two distinguishing features of the small RNAs libraries are the populations and distributions. The composition of small RNAs often reflects roles of different categories of small RNAs in a particular tissue or species or associated biogenetic machines. Since most of the small RNAs with known functions are 20-24 nt long, we only investigated the distribution of small RNAs of 18-25 nt in the three libraries (Figure 1; Figure 2). In the three ripening stages, the 24-nt size class was the overall most abundant class of sRNAs (Figure 2), which was consistent with that of Arabidopsis [36] and rice [37], but different from that of wheat, Chinese yew and grapevine [38-40]. The higher percentage of 24-nt small RNAs in tomato fruit may reflect the complexity of the tomato genome because 24-nt siRNAs are known as predominant heterochromatin-associated sRNAs [41]. The 21-24nt sRNAs exhibited a peak at breaker stage, and showed a sharp reduction at the red ripening stage, suggesting potential roles in ripening process.

### Identification of known and novel miRNAs in tomato fruit

**Figure 3:**
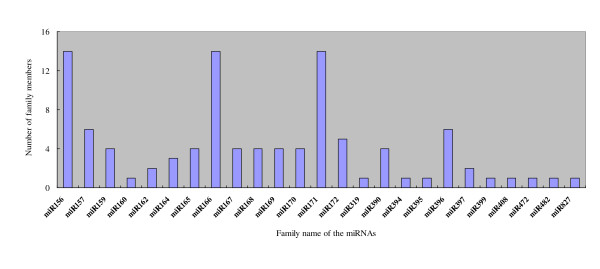
**Conserved miRNAs and their family numbers in tomato fruit**. The members of each family were different, the miR156, miR166 and miR171 had more than ten members, in the contrary, miR160, miR319, miR394, miR395, miR399, miR408, miR472, miR482, miR827 had only one member in their corresponding family.

Conserved miRNAs were found in many plant species and have important functions in plant development and stress response [8]. To identify the conserved miRNAs in tomato, sRNA sequences obtained by deep sequencing were compared with the currently known mature plant miRNAs in miRBase [42]. After Blastn searches and further sequence analysis, a total of 103 conserved miRNAs (Figure 3), belonging to 24 miRNA families were identified and all of which can be consulted in two important database (http://www.mirbase.org/; http://ted.bti.cornell.edu/cgi-bin/TFGD/sRNA/miRNA.cgi).

**Figure 4:**
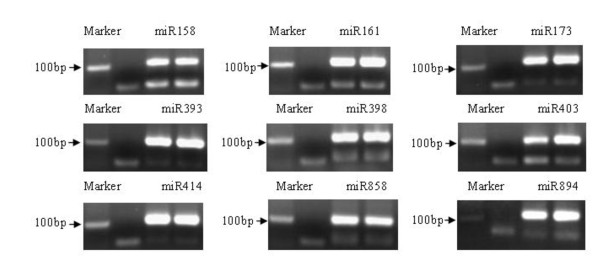
**The validation of nine identified non-conserved miRNAs in tomato fruit**. The total length of the RTS primer and the miRNA sequence was about 110 bp, the nine non-conserved families were validated, the right two lanes were the miRNAs and the second left lane was the negative control.

In addition to the conserved miRNAs, there are known miRNAs that are not-conserved, but are found in only one or a few plant species such as *Arabidopsis *or *Physcomitrella patens *[8,23]. Ten members belong to 9 non-conserved miRNAs (miR158, miR161, miR173, miR393, miR398, miR403, miR414, miR858, miR894) were present in our data sets at a low abundance, with the exception of miR403 that were represented by a relative high number (Table 2). All of the non-conserved miRNAs were validated by the construction of the small RNA cDNA library method (Figure 4) [43].

**Table 2:** Conserved and non-conserved miRNAs and their expression profiles at different tomato fruit ripening stages

MiRNA family	References	Relative number of reads found in tomato		
		
		Mature green	Breaker stage	Red ripe
Conserved miRNAs				
miR156	Zhang J *et al*, 2008; Yin Z *et al*, 2008; Moxon S *et al*, 2008.	115.99	31.95	15.14
miR157	Zhang J *et al*, 2008; Yin Z *et al*, 2008; Moxon S *et al*, 2008.	349.64	154.19	137.34
miR159	Pilcher *et al*, 2007; Zhang J *et al*, 2008; Moxon S *et al*, 2008.	59.89	48.06	82.55
miR160	Pilcher *et al*, 2007; Yin Z *et al*, 2008; Moxon S *et al*, 2008.	46.06	30.28	1.44
miR162	Pilcher *et al *l, 2007; Yin Z *et al*, 2008; Moxon S *et al*, 2008.	878.08	929.27	167.99
miR164	Pilcher *et al*, 2007; Moxon S *et al*, 2008	996.58	496.45	188.53
miR165	Moxon S *et al*, 2008	38.94	24.73	3.60
miR166	Pilcher *et al*, 2007; Moxon S *et al*, 2008	2750.64	2154.69	523.78
miR167	Zhang J *et al*, 2008; Yin Z *et al*, 2008; Moxon S *et al*, 2008.	265.06	209.19	8.29
miR168	Pilcher *et al*, 2007; Yin Z *et al*, 2008; Moxon S *et al*, 2008.	4967.40	3130.92	62.36
miR169	Zhang J *et al*, 2008; Yin Z *et al*, 2008; Moxon S *et al*, 2008.	0.84	0	0
miR171	Pilcher *et al*, 2007; Zhang J *et al*, 2008; Moxon S *et al*, 2008.	19.68	53.61	2.16
miR172	Zhang J *et al*, 2008; Yin Z *et al*, 2008; Moxon S *et al*, 2008; Itaya *et al*, 2008.	2993.09	2733.37	798.11
miR319	Zhang J *et al*, 2008; Yin Z *et al*, 2008; Moxon S *et al*, 2008.	0.42	0	0
miR390	Moxon S *et al*, 2008; Itaya *et al*, 2008.	97.98	46.67	2.16
miR394	Moxon S *et al*, 2008.	6.70	4.44	2.16
miR395	Zhang J G *et al *2008; Moxon S *et al*, 2008.	0.41	0	0
miR396	Moxon S *et al*, 2008.	1300.58	2175.81	334.89
miR397	Moxon S *et al*, 2008.	0.42	0	0
miR399	Zhang J *et al*, 2008; Yin Z *et al*, 2008; Moxon S *et al*, 2008.	44.38	17.50	1.08
miR408	Pilcher *et al*, 2007, Moxon S *et al*, 2008	0.42	0.28	0
miR472	Itaya *et al*, 2008; Moxon S *et al*, 2008.	19.26	12.78	0.36
miR482	Pilcher *et al*, 2007.	1.67	2.51	0
miR827	http://ted.bti.cornell.edu/cgi-bin/TFGD/sRNA/miRNA.cgi	2.09	6.39	14.78
Non-conserved miRNAs				
miR158		2.93	1.67	0
miR161		0.42	0	0
miR173		0.41	0	0
miR393		0.42	0	0
miR398		0.40	0	0
miR403		210.20	138.91	27.04
miR414		0.42	0	0
miR828	Moxon S *et al*, 2008.	0	0	0
miR858	Moxon S *et al*, 2008.	5.19	12.31	1.22
miR894	Moxon S *et al*, 2008.	10.05	5.00	0.36

**Figure 5:**
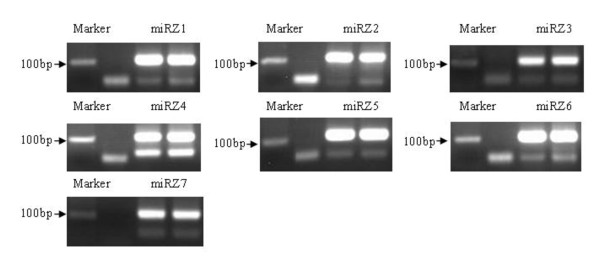
**The validation of seven novel miRNAs in tomato fruit**. The total length of the RTS primer and the miRNA sequence was about 110 bp, the novel seven miRNA families were validated, the right two lanes were the miRNAs and the second right lane was the negative control.

**Figure 6:**
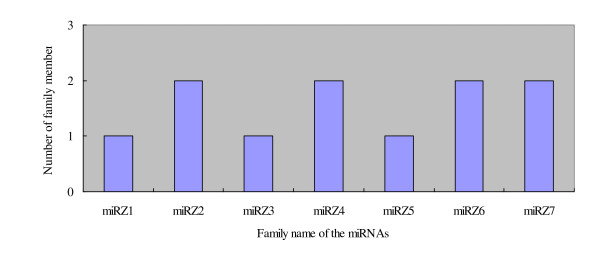
**The member distribution of the seven novel miRNA families in tomato fruit**. Seven novel miRNA families were found in the libraries, miRZ1, miRZ3 and miRZ5 had only one member, however, and the rest families (miRZ2, miRZ4, miRZ6, miRZ7) had two members.

A number of criterions are used for evaluating whether a small RNA is a genuine miRNA, such as formation of a stable hairpin structure, starting with a 5' uridine, lower minimal free energies (MFEs) for hairpin structure of its precursors, and detection of miRNA*s [44,45]. By using these rules, we identified 11 novel miRNAs belonging to 7 families (named miRZ1-miRZ7, Figure 5, Figure 6, additional file 1, 2). Except for miRZ6, most of the novel miRNAs were in low copies. The possible reason is that the encoding genes are not broadly conserved and expressed at much lower levels [46].

### Prediction of the targets of identified miRNAs in tomato fruit

Target prediction for miRNAs is straightforward because it is assumed that most of them match their targets with almost perfect complementarity [9,47]. The putative target genes for all identified conserved and non-conserved miRNAs were searched by using the web-based computer psRNA Target Server (http://plantgrn.noble.org/psRNATarget/) which identifies putative targets regulated at post-transcriptional or translational levels.

**Figure 7:**
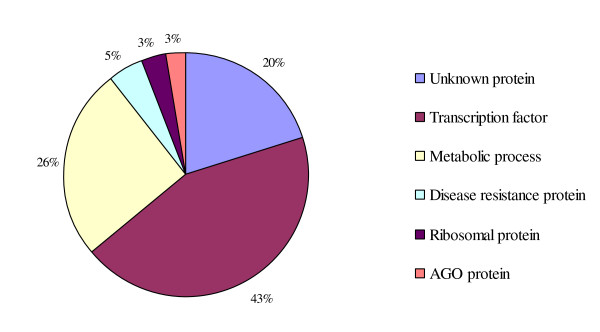
**Distribution of the predicted target genes of known miRNAs in tomato fruit**. The targets of the known miRNAs were mainly divided into three types, almost half of them were transcription factors, and nearly one third of them were involved in metabolic process, one fifth of the targets were unknown, in addition, several targets were predicted to participant in disease resistance and their own biosynthetic pathway.

Most conserved miRNA targets that are conserved across several plant species, including Arabidopsis [48], rice [49], grape [50], poplar [51] and wheat [52]. And most miRNA families have multiple target sites, suggesting that these miRNAs are functionally divergent. In our study, 152 targets of conserved and non-conserved miRNAs were predicted (Figure 7). The majority of such targets are various transcriptional factors including SBP (miR156), MYB (miR159, miR319, miR172), NAM (miR164), and MADS-Box (miR396) that regulate plant development [53] or phytohormone signal transduction [54]. Other conserved miRNAs targets include F-box protein (miR394, miR414), ATP sulfurylase (miR395), Pectate Lyase (miR482), endo-1, 4-beta- glucanase (miR396), Laccase (miR397), all of which are involved in regulation of metabolic processes. Several targets of the miRNAs are AGO protein (miR168, miR403) which regulate their own biosynthesis pathway. In addition, targets of the conserved miRNAs include disease resistance proteins (miR472, miR482) which are related to pathogen resistance [33,34]. Furthermore, ACS and EIN2, which are involved in ethylene biosynthesis and signal transduction [17], are putative targets of miR159 and miR828, respectively.

The targets of the novel miRNAs identified in our library were predicted, most of which were unknown (Additional file 1). MiRZ1 was predicted to be related to disease response, MiRZ6 was predicted to be involved in virus infection response. Interestingly, a target of miRZ7 is beta-galactosidase which is an important enzyme affects fruit softening. Another member's target of miRZ7 family is starch synthase which participates in starch biosynthesis, including transient starch [55].

### Expression profiles of the miRNAs involved in tomato fruit ripening

**Figure 8:**
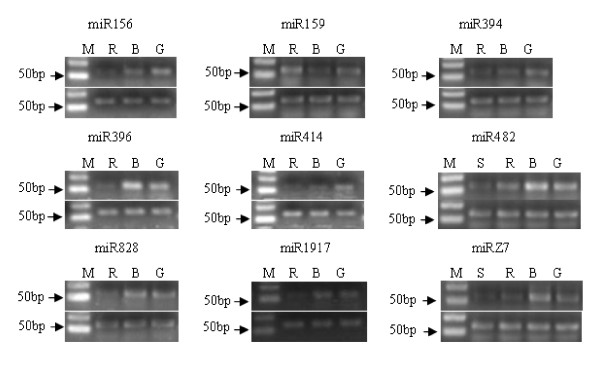
**The expression patterns of the known and novel miRNAs in tomato fruit**. MiR156 and miR394 were down regulated in the fruit ripening, miR159 showed down regulation in the breaker stage, while, miR396 showed a obvious increase in the breaker stage, miR828 and miR1917 were down regulated in the red ripe stage, miR482 and miRZ7 showed down regulation in the red and softening ripe stage. (Annotation: M-Marker; R-red ripe stage; B-The breaker stage; G- green ripe stage, U6 was used as the reference gene).

It has been shown that high throughput sequencing provides an alternative way to gain the genome-wide transcript profiles of miRNAs abundance [56] and allows us to determine the abundance of various miRNA families and even to distinguish different members of a given family of one organism. To elucidate the potential roles of miRNAs in tomato fruit ripening process, we profiled the expression levels of known and novel miRNAs. We computed the normalised counts of known miRNA sequences, plotted them across the three ripening stages and validated some results by stem-loop RT-PCR (Figure 8).

The abundance of most known miRNAs decreased during fruit ripening, and some were found only in the mature green stage (Table 2). A few known miRNAs were expressed at high levels in fruit, such as miR157, miR162, miR164, miR166, miR168, miR172, miR396. On the other hand, other miRNAs, such as miR161, miR173, miR393, miR397, miR398 and miR414, were expressed at relatively lower levels and can only be detected in mature green stage, Particularly, the expression levels of miR159 decreased at the breaker stage and increased at red ripening stage compared with that of the mature green stage which reveals its probable specific roles in fruit ripening or ethylene pathway, as one of the predicted targets is 1-aminocyclopropane -1-carboxylate synthase which is a crucial enzyme in ethylene biosynthesis [17]. Expression of another miRNA, miR396, increased from mature green to breaker stage, then dropped sharply in the red stage. Interestingly, targets of miR396 include MADS-Box protein and endo-1, 4-beta-glucanase, both of which are involved in fruit ripening and softening process [57]. Moreover, 3 miRNAs (miR394, miR414, and miR482) predicted to have targets related to fruit ripening and softening and ethylene response were validated (Figure 8).

Colorless non-ripening (CNR), a member of the squamosa-promoter binding protein (SBP) family that was shown to be involved in fruit ripening [58], is targeted by miR156 [31,39]. The expression levels of miR156 also decreased during fruit ripening (Table 2 and Figure 8). Two miRNAs, miR828 and miR1917, whose targets are ethylene-insensitive 2 (EIN2) and serine/threonine protein kinase (CTR1) which are vital regulators in ethylene signal transduction[17,59], reported previously [26] and not shown in our sequencing results, showed reduced expression during fruit ripening (Figure 8). A novel miRNA (miRZ7) expression levels were also analyzed which was predicted to relate to fruit softening.

Overwhelming the majority of the stem-loop RT-PCR results was in agreement with sequencing results. Only two miRNAs (miR414, miRZ7) were exception which could be detected by stem-loop RT-PCR but were not detected or barely detectable by sequencing. This could be due to cross amplification of other highly homologous small RNA members or biases occurred during library generation or sequencing in some samples.

### Expression profiles of the miRNAs responses to exogenous ethylene in tomato fruit

**Figure 9:**
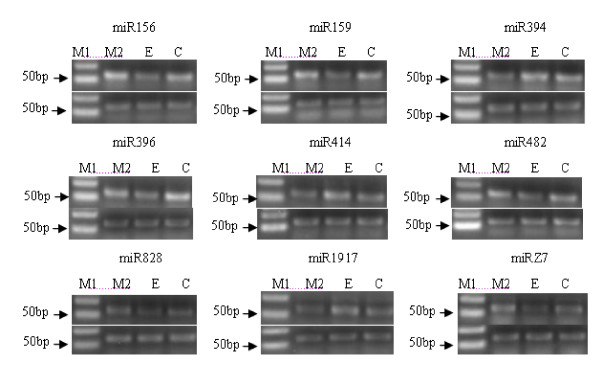
**The expression patterns of known and novel miRNAs after exogenous ethylene treatment in tomato fruit**. Three miRNA families (miR394, miR414 and miR1917) were down regulated, in the contrary, four miRNA families (miR156, miR159, miR396, miR482 and miRZ7) were up-regulated, and however, the miR828 had no obvious change. (Annotation: M1-Marker; M2-1-methylcycloproene treatment; E-exogenous ethylene treatment; C-control, U6 was used as the reference gene).

Ethylene is a regulator of various physiological and morphological responses, including seedling development, leaf and flower senescence, induction of fruit ripening, floral sex determination, resistance to pathogen infection, and adaptation to stress conditions [60-63]. Tomato has proven to be a highly successful model system for fruit development and ripening. And the role of ethylene during fruit ripening has been most thoroughly studied in tomato fruit [15,16]. In order to find out the relationships between ethylene treatment and miRNAs expression, exogenous ethylene and 1-methylcycloproene were used to treat the mature green fruits. The expression levels of a number of known and novel miRNAs were tested by stem-loop RT-PCR. Three miRNAs (miR394, miR414, miR1917) were down regulated; five miRNAs (miR156, miR159, miR396, miR482, miRZ7) were up regulated, while one miRNA (miR828) whose target is EIN2 was not affected, by exogenous ethylene treatment (Figure 9).

## Discussions

### High-throughput sequencing of short RNAs in tomato

High-throughput sequencing has been used to study miRNAs at the whole genome level in several model plant species, including Arabidopsis [23], rice [25], and wheat [64]. The successful application of high-throughput sequencing technology to systemically identify plant miRNAs in recent years has greatly advanced our knowledge on the functions of miRNAs in plants.

The composition of small RNAs often reflects roles of different kinds of small RNAs in a specific tissue or species. Several plant species, such as *Pinus cordata*, *Populus balsamifera *and *grapevine *were shown to contain substantially more 21-nt than 24-nt sRNAs [37,39,65]. However, we observed an unusually high level of 24-nt sRNAs compared with the 21-nt class (Figure 1; Figure 2), as in several other plant miRNAs studies [23,36,37,66]. The number of 24-nt small RNAs was almost three times that of the 21-nt class, such a high percentage of 24-nt small RNAs may reflect the complexity of the tomato genome because 24-nt siRNAs are known to be involved in heterochromatin modification, especially for genomes with high content of repetitive sequences [67,68].

Plant MIR genes are possible to arise from gene duplication events which then evolved by random mutations into short, imperfectly paired hairpins [46,69]. Those non-conserved miRNAs are believed to be evolutionarily recent and generally represented by single copy MIR genes [8]. Recently, many non-conserved miRNAs were reported in several species [36,37,64,66]. Some of these non-conserved miRNAs are not found in other species/families and several non-conserved miRNAs are found in different phylogenetic families. We also found nine non-conserved miRNAs in our database, although most of them were present at a very low level. However, the low number of sequence reads does not rule out that they could be expressed at a high level in specific cells. Few non-conserved miRNAs were expressed at a relatively high level, suggesting that they may represent an intermediate status between the deeply conserved and the less conserved miRNAs.

New species-specific miRNAs are considered to be young miRNAs that have evolved recently, and are often expressed at a lower level than conserved miRNAs, as was reported for Arabidopsis and wheat [23,36,38]. This observation is true for many new tomato miRNAs identified here. However, few new miRNAs were expressed at a high level in a tissue-specific manner (miRZ6). In some cases we observed inconsistency between the level of miRNAs identified by Solexa sequencing and experimental verification. It is possible that during library generation or sequencing some bias could occur for certain sequences in some samples.

In contrast to the 454 pyrosequencing sequencing in tomato previously [39], more reads were obtained in each of our three libraries, and miRNAs at different fruit ripening stages were additionally analyzed in our study. In our database, most conserved miRNAs have been detected, including 33 conserved and non-conserved miRNA families, which cover all the published miRNAs in the two databases for tomato miRNAs (http://www.mirbase.org/, http://ted.bti.cornell.edu/cgi-bin/TFGD/sRNA/miRNA.cgi).

Dalmay and colleagues studied the correlation between the short RNAs and fleshy fruit development using deep sequencing technology [70]. However, mostly sRNAs were analyzed, which is different from our miRNAs study. But they reported several miRNAs, and validated one novel miRNA related to the glutamate accumulation which contributes to the tomato fruit taste.

### Targets of known and novel miRNAs in tomato fruit

Target prediction is necessary for assessing miRNAs' putative functions. Currently, the most efficient tool available for this is the bioinformatics approach facilitated by the high degree of homology between miRNA and its target sequences in plants [71]. Our analysis reveals that most of the predicted targets in tomato have a conserved function among a variety of plant species [72]. Consistent with previous reports, the largest of these targets in tomato fruit were plant-specific transcription factors, such as AP2, NAC, SBP and the ARF family [53]. The second largest targets encoded a range of different proteins implicated in a variety of metabolic processes, such as ATP sulfurylase, Pectate Lyase, endo-1, 4-beta-glucanase, Laccase. In addition, functions of several targets are largely unknown.

Although many conserved miRNA targets were predicted, only a limited number of miRNA targets were identified experimentally [31,39]. Several miRNAs slice their targets and can be validated by RACE. Alternatively, other miRNAs inhibit target gene expression through translational arrest, such as miR156 and miR172, which have been shown to regulate their target genes (SBPs and AP2) predominantly by inhibiting their translation [73-76]. Indeed, genetic and biochemical evidence is accumulated that plant miRNA-guided silencing has a widespread translational inhibitory component [2,77].

The novel tomato miRNAs target different genes with a wide variety of predicted functions. It may be worthy to note that those miRZ6 targets six members of disease resistance protein (CC-NBS-LRR class), which are likely to be involved in host-pathogen interactions. Surprisingly, one target of miRZ7 is beta-galactosidase which is a crucial enzyme for fruit softening, which is beneficial for clarify the cooperating roles in freshly fruit ripening and senescence. The target of another member in miRZ7 family is starch synthase which participates in regulating starch biosynthesis (Additional file 1) [55].

### Fruit ripening and softening related miRNAs

Functional analysis has been carried out only for role of a few tomato miRNAs morphological development [28-30]. Conserved and non-conserved miRNAs regulate genes involved in fleshy fruit development were reported previously [39]. A target gene of miR156 belong to squamosa-promoter binding protein (SBP) family called CNR was validated, which plays pivotal roles in fruit ripening [57]. Over-expression of miR156 can cause the fruit red colour slightly lighter than the wild type, which was thought that the expression of miR156 and CNR overlaps only partially and the function of the miRNA is to suppress CNR expression in specific cell types. CNR mRNA is up-regulated in fruit at the breaker stage [57], coincidently, our sequence results showed a negative correlation as expected. Two important targets of miR396 are endo-1, 4-beta-glucanase and MADS-box protein, both of which are important regulators in fruit ripening and softening [78,79]. Our sequencing results showed that the abundance of miR396 peaked at the breaker stage and decreased sharply in red ripening stage suggesting its possible function in fruit ripening. A target of miR482 is pectate lyase which is an important enzyme in fruit softening [80,81]. Meanwhile, a novel miRNA, whose target is beta-galactosidase, which is also a key softening related enzyme [82,83]. The expression levels of the two miRNAs were also coinciding with the assumption including the softening ripening stage (Figure 8).

Intriguingly, an in-depth study of the tomato transcriptome and proteome unraveled the regulation mechanisms during fruit ripening stages. The transcriptional regulation is mostly responsible for global reductions in plastid gene expression prior to or at the onset of fruit formation whereas post-transcriptional events become predominant at the breaker stage [84,85]. These post-transcriptional events did not lead to significant reductions in mRNA levels, although mRNA stability was reported to be modestly affected for some genes [84]. miRNAs and their targets have been shown to regulate in translational inhibition way in a fairly large fraction [2].

### Ethylene biosynthesis and signal transduction related miRNAs in tomato

Recent researchers have witnessed the great progress in the area of identification of interaction between miRNAs pathways and phytohormone responses, which improves our understanding of mechanism of plant development controlled by miRNAs and hormone action to a large extent. One of the most important phytohormone is ethylene which plays vital roles during all stages of the plant life cycle, functioning through seed germination to ripening and various abiotic stress conditions [86]. MiR159 and miR394 were reported to be associated with ethylene in rice [87]. Most targets of miR159 are MYB, and a novel target not related to MYB in tomato recently [30]. Another important target of miR159 is 1-aminocyclopropane-1-carboxylate synthase which plays vital roles in ethylene biosynthesis [17]. As a typical climacteric plant, abundance ethylene was synthesized at the breaker stage approximately, and the expression level of miR159 was suppressed which match our results well. MiR394 and miR414 also target F-box family proteins which are important participants in the signal transduction pathways of different plant hormones [88,89]. The stability of the ethylene signaling regulators EIN2 and EIN3 are modulated by the F-box proteins ETP1/2and F-box proteins EBF1/2 respectively. Ethylene Insensitive 2 (EIN2), a target of miR828, is the key positive regulator of ethylene signal transduction and other hormones such as abscisic acid, auxin, cytokinin and jasmonate and thus may represent a point of crosstalk between multiple hormone signaling pathways [90-92]. CTR1 is a negative regulator of ethylene response that likely interacts directly with receptor molecules to form a signaling complex [93,94]. As with the ethylene receptors, all tissues evaluated express *CTR1 *genes and their mRNAs are differentially accumulated depending on tissue. LeCTR1 induction is associated with tissues at stages of development associated with increased ethylene, including fruit ripening and is a validated target of miR1917 [95]. Exogenous ethylene can influence the miRNAs expression patterns, most of which shown negative correlation to their targets well except for miR828, was not affected by exogenous ethylene treatment (Figure 9). The most likely reason was that miR828 affected this target slightly and we cannot catch the discrepancy.

## Conclusions

We have used a combination of high throughput sequencing and molecular biology approaches to explore the involvement of miRNAs during fruit ripening process. Total 33 conserved and non-conserved miRNA families and 7 novel miRNA families were indentified. The expression profiles of miRNAs at different ripening stages were analyzed and validated simultaneously, most of which were in agreement with the sequencing results. The influences of exogenous ethylene on miRNAs expression were also studied. These findings provide valuable information for further functional verification of miRNAs in tomato fruit ripening and ethylene response.

## Methods

### RNA analysis

Tomato samples from three ripening stages (mature green, breaker, and red-ripening stage) of *Solanum lycopersicum 'Ailsa Craig' *were used to prepare for the high throughout sequencing. Total RNAs were extracted using TRIzol reagents. For each sample, the 18-30nt small RNAs were ligated with 5'- and 3'- RNA adapter by T4 RNA ligase after being purified by polyacrylamide gel electrophoresis. The RNAs were subsequently transcribed to single-stranded cDNAs using Superscript II reverse transcriptase. Thereafter the cDNAs were used as templates for double-stranded cDNA synthesis by PCR amplification using primers that anneal to adapters. The purified cDNAs were sequenced on an Illumina-Solexa 1 G Genetic Analyzer (BGI).

### Bioinformatics analysis

The adapter sequences of the Solexa sequencing results were removed. And sequences larger than 30nt and smaller than 18nt were discarded. All high quality sequences were considered as significant and further analyzed. Small RNA sequences were mapped to tomato genome (ftp://ftp.sgn.cornell.edu/tomato_genome/ITAG_devel_release/). rRNAs, tRNAs, snRNAs and snoRNAs were removed from the matched sequences through BLASTn search using NCBI Genebank database (http://www.ncbi.nlm.nih.gov/blast/Blast.cgi/). Mismatches were not allowed in the above two approaches. The unique sequences left were aligned with known miRNAs from miRBase 10.0 (http://www.mirbase.org/) [42]. The potential novel miRNAs were analyzed using mireap. Parameters were set based on a previous plant miRNAs study [45]. The court number was normalized as transcript per million (TPM). Target predictions were performed using the psRNATarget (http://plantgrn.noble.org/psRNATarget/) [64].

### Exogenous ethylene and 1-Methylcycloproene treatment

*Solanum Lycopersicum *(*Ailsa Craig*) plants were grown in soil under standard greenhouse conditions. Mature green fruits were harvested and divided into three groups, the first group was treated with 50 μl/L of ethylene for 6 h, the second group was treated with 0.5 μl/L 1- methylcyclopropene (1-MCP) for 24-h, and the third group was used as a control. After treatments, samples were frozen immediately in liquid nitrogen and stored at -80°C until RNA extraction [96].

### MicroRNAs Detection

Small RNA samples from above fruit samples were isolated using the miRNA isolation kit (Bioteke) according to the manufacturer's instructions. Small RNA samples were polyadenylated at 37°C for 60 min in a 50-μl reaction volume containing 0.5 μg RNA and 1.5 U poly (A) polymerase (Ambion). The reaction product was diluted to 300 μl. An equal volume of acid-phenol:chloroform was added, mixed and centrifuged. The aqueous phase was carefully removed and transferred to a new tube. Two volume of ethanol and one-tenth volume of sodium acetate (3 M, pH 5.2) were added and mixed thoroughly. The mixture was allowed to precipitate at -20°C for 2 h and then centrifuged at 12000 rpm for 20 min. The supernatant was discarded and the pellet was dissolved with 20 μl RNA-free water. To generate a small RNA cDNA (srcDNA) library, 20 μl of the tailed RNA and 1 μl of RTS primer were mixed in a 26-μl reaction volume, incubated at 65°C for 10 min, and annealed at 4°C for 20 min. Reverse transcription was carried out with reverse-transcriptase (Invitrogen) at 50°C for 60 min. Finally, the reverse transcriptase was inactivated by incubation at 70°C for 15 min. A small RNA-specific primer and a universal reverse primer were used for amplification of individual small RNAs (Additional file 3). The annealing temperature was adjusted according to the T_m _of individual small RNAs. After PCR, an aliquot of the PCR products was analyzed on a 2.5% agarose gel [43].

### Differential expression analysis of microRNAs

Total RNA was extracted from the samples using TRIzol solution (Trans, Beijing, China) and treated with RNase-free DNase I (Promaga, Beijing, China). First strand cDNA was synthesized using total RNA and reverse transcriptase (Promaga, Beijing, China). Expression levels of mature miRNAs were analyzed by Semi-quantitative RT-PCR stem-loop method [97-100]. A stem-loop containing RT primer with its 5'-end complementary to target miRNA's last 6-nt at 3'-end was designed. Reverse transcription was performed at 16°C for 30 minutes, followed by 60 cycles of pulsed RT at 30°C for 30 seconds, 42°C for 30 seconds and 50°C for 1 second. Semi-quantitative RT-PCR was performed using a forward primer containing the 5' part sequence of miRNA and a universal primer complementary to the stem-loop part of RT primer at 94°C for 2 min, followed by 21 cycles of 94°C for 15 s and 60°C for 1 min. The reaction products (about 60 bp) were analyzed by electrophoresis on a 2.5% agarose gel in 1× TAE. The primers used in this study were listed in the Additional file 4.

## Authors' contributions

BZ Z and JH Z design the experiments, JH Z conducted the experiments, YZ M, LH C, YB L, DQ F, Y Z and BZ Z participate in bioinformatics analysis, YX W and Z J participate in RNA extraction, JH Z wrote the paper. All authors read and approved the final manuscript.

## Supplementary Material

Additional file 1 **Novel miRNAs sequences and their potential targets in tomato fruit**. Seven novel miRNAs families identified in tomato fruit and their sequence information and their predicted target proteins. Click here for file

Additional file 2 **The detailed information of seven novel miRNAs in tomato fruit**. The precursor sequence of the identified seven novel miRNAs and part of the miRNA*sequence. Click here for file

Additional file 3 **Primers for validation of non-conserved and novel miRNAs in tomato fruit**. The universal and specific primers sequence information for the non-conserved and novel miRNAs validation. Click here for file

Additional file 4 **Primers for stem-loop RT-PCR analysis of miRNAs in tomato fruit**. The universal and specific stem-loop primers sequence information for the validation of the miRNAs expression files. Click here for file
