# Supplementary material for: Sculpting the maturation, softening and ethylene pathway: The influences of microRNAs on tomato fruits
Source: BMC Genomics. 2012 Jan 9;13:7. doi: 10.1186/1471-2164-13-7 (PMC3266637; doi:10.1186/1471-2164-13-7)
Supplement: Additional file 1 — Novel miRNAs sequences and their potential targets in tomato fruit. Seven novel miRNAs families identified in tomato fruit and their sequence information and their predicted target proteins. [file 1471-2164-13-7-S1.DOC]

**Additional file1:**

Tab 1. Novel miRNAs sequences and their potential targets in tomato fruit

| **Family** | **Sequence(5’-3’)** | **Target gene** | **Target Protein** | **Target function** |
| --- | --- | --- | --- | --- |
| miRZ1 | ATTTTTGAAGAGTCCGAGCA | [SGN-U317189](http://plantgrn.noble.org/psRNATarget/getseq.do?sessionid=1316797078891431&source=target&seqID=SGN-U317189) | leucine-rich repeat family protein | disease resistance |
| miRZ2 | TCAATGCTACATACTCATCCC | [SGN-U326896](http://plantgrn.noble.org/psRNATarget/getseq.do?sessionid=1316797657114898&source=target&seqID=SGN-U326896) | expressed protein | Unknown |
| miRZ3 | AGCTGCTGACCTATGGATTCC | [SGN-U344491](http://plantgrn.noble.org/psRNATarget/getseq.do?sessionid=1316797738570918&source=target&seqID=SGN-U344491) | Unknown | Unknown |
| miRZ4 | TCCTGCCTTGCATCAACTGAAT | [SGN-U327227](http://plantgrn.noble.org/psRNATarget/getseq.do?sessionid=1316797819914699&source=target&seqID=SGN-U327227) | Unknown | Unknown |
| miRZ5 | TAGGGTGTCGAGTTGAGGAGA | [SGN-U318124](http://plantgrn.noble.org/psRNATarget/getseq.do?sessionid=1316797891177842&source=target&seqID=SGN-U318124) | chloroplast ADP, ATP translocase 1 | metabolic process |
| miRZ6 | TGAATCCTTCGGCTATCCATAA | [SGN-U330478](http://plantgrn.noble.org/psRNATarget/getseq.do?sessionid=1316798075066894&source=target&seqID=SGN-U330478) | disease resistance protein | disease resistance |
| miRZ7 | TGTTTCTCGTGAATCCTTCGGC | [SGN-U312682](http://plantgrn.noble.org/psRNATarget/getseq.do?sessionid=1316798165822052&source=target&seqID=SGN-U312682) | beta-galactosidase | Fruit softening |
