# Supplementary material for: Sculpting the maturation, softening and ethylene pathway: The influences of microRNAs on tomato fruits
Source: BMC Genomics. 2012 Jan 9;13:7. doi: 10.1186/1471-2164-13-7 (PMC3266637; doi:10.1186/1471-2164-13-7)

**Additional file2:**

**Fig 1:** The detailed information of seven novel miRNAs in tomato fruit

miRZ1


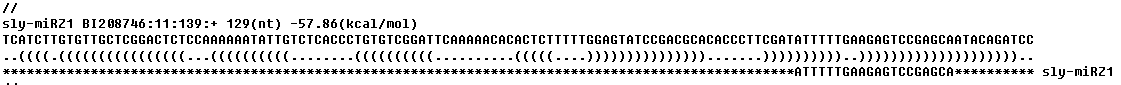


miRZ2


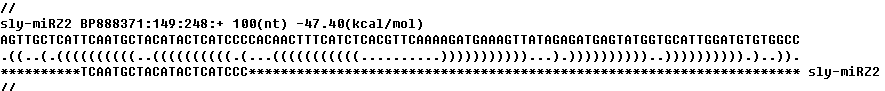


miRZ3


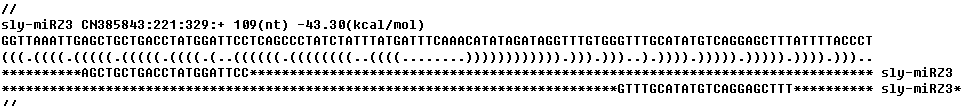


miRZ4


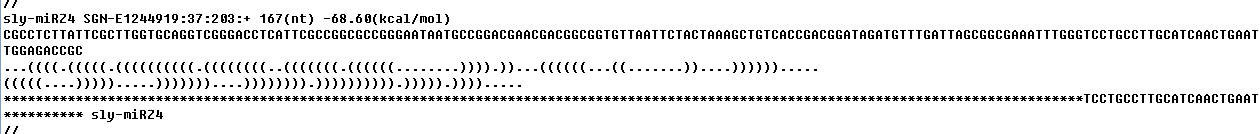


miRZ5


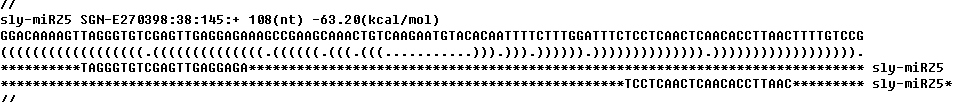


miRZ6


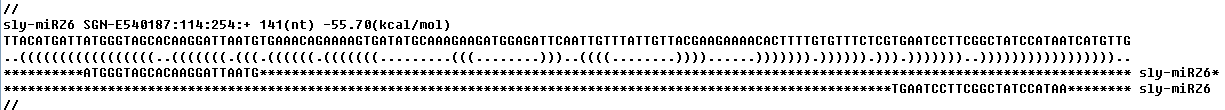


miRZ7


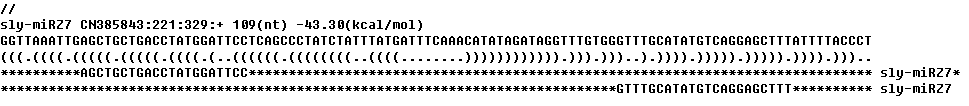

Supplement: Additional file 2 — The detailed information of seven novel miRNAs in tomato fruit. The precursor sequence of the identified seven novel miRNAs and part of the miRNA*sequence. [file 1471-2164-13-7-S2.DOC]
