# Supplementary material for: Sculpting the maturation, softening and ethylene pathway: The influences of microRNAs on tomato fruits
Source: BMC Genomics. 2012 Jan 9;13:7. doi: 10.1186/1471-2164-13-7 (PMC3266637; doi:10.1186/1471-2164-13-7)
Supplement: Additional file 3 — Primers for validation of non-conserved and novel miRNAs in tomato fruit. The universal and specific primers sequence information for the non-conserved and novel miRNAs validation. [file 1471-2164-13-7-S3.DOC]

**Additional file3:**

Tab 2. Primers for valitation of non-conserved and novel miRNAs in tomato fruit

| **Primer** | **Sequence(5’-3’)** |
| --- | --- |
| RTS | AGCTCGAGGCAGGCGACATGGCTGGCTAGTTAAGCTTGGTACCGAGCTCGGATCCACTAGTCCTTTTTTTTTTTTTTTTTTTTTTTTTVN |
| Universal | AGCTCGAGGCAGGCGACATGG |
| miR158 | TCCCA AATGTAGACA AAGCA |
| miR161 | TTGAA AGTGACTACATCGGGG |
| miR173 | TCGCT TGCAG AGAGA AATCACA |
| miR393 | TCCAA AGGGA TCGCATTGATCC |
| miR398 | TATGT TCTCAGGTCG CCCCTG |
| miR403 | CTAGATTCACGCACAAGCTCG |
| miR414 | CATCATCATCATCATCGTCA |
| miR858 | CTCATTGTCTGTTCGACCTTG |
| miR894 | CGTTTCACGTCGGGTTCACC |
| miRZ1 | ATTTTTGAAGAGTCCGAGCA |
| miRZ2 | TCAATGCTACATACTCATCCC |
| miRZ3 | AGCTGCTGACCTATGGATTCC |
| miRZ4 | TCCTGCCTTGCATCAACTGAAT |
| miRZ5 | TAGGGTGTCGAGTTGAGGAGA |
| miRZ6 | TGAATCCTTCGGCTATCCATAA |
| miRZ7 | TGTTTCTCGTGAATCCTTCGGC |
