# Supplementary material for: Sculpting the maturation, softening and ethylene pathway: The influences of microRNAs on tomato fruits
Source: BMC Genomics. 2012 Jan 9;13:7. doi: 10.1186/1471-2164-13-7 (PMC3266637; doi:10.1186/1471-2164-13-7)
Supplement: Additional file 4 — Primers for stem-loop RT-PCR analysis of miRNAs in tomato fruit. The universal and specific stem-loop primers sequence information for the validation of the miRNAs expression files. [file 1471-2164-13-7-S4.DOC]

**Additional file4:**

Tab.3 Primers for stem-loop RT-PCR analysis of miRNAs in tomato fruit

| **MicroRNA** | **primer** | **Sequence(5’-3’)** |
| --- | --- | --- |
| miR156 | RT-Primer | GTCGTATCCAGTGCAGGGTCCGAGGTATTCGCACTGGATACGAC GTGCTC |
|  | Forward Primer | GCGGCGGTGACAGAAGAGAGT |
| miR159 | RT-Primer | GTCGTATCCAGTGCAGGGTCCGAGGTATTCGCACTGGATACGAC TAGAGC |
|  | Forward Primer | CGGCGGTTTGGATTGAAGGGA |
| miR394 | RT-Primer | GTCGTATCCAGTGCAGGGTCCGAGGTATTCGCACTGGATACGAC GGAGGT |
|  | Forward Primer | GCGGCGGTTGGCATTCTGTCC |
| miR396 | RT-Primer | GTCGTATCCAGTGCAGGGTCCGAGGTATTCGCACTGGATACGAC CAGTTC |
|  | Forward Primer | GCGGCGTTCCACAGCTTTCTT |
| miR414 | RT-Primer | GTCGTATCCAGTGCAGGGTCCGAGGTATTCGCACTGGATACGAC TGACGA |
|  | Forward Primer | GCGGCGGCATCATCATCATCA |
| miR482 | RT-Primer | GTCGTATCCAGTGCAGGGTCCGAGGTATTCGCACTGGATACGAC  TAGGAA |
|  | Forward Primer | GCGCGTTTCCAATTCCACCCA |
| miR828 | RT-Primer | GTCGTATCCAGTGCAGGGTCCGAGGTATTCGCACTGGATACGAC TGG AAT |
|  | Forward Primer | CGGCGGTCTTGCTTAAATGAGT |
| miR1917 | RT-Primer | GTCGTATCCAGTGCAGGGTCCGAGGTATTCGCACTGGATACGAC ACTTTA |
|  | Forward Primer | GCGGCGGATTAATAAAGAGTGC |
| miRZ7 | RT-Primer | GTCGTATCCAGTGCAGGGTCCGAGGTATTCGCACTGGATACGAC AAGCCG |
|  | Forward Primer | CGGCGGTGTTTCTCGTGAATCC |
|  | Universal primer | GTGCAGGGTCCGAGGT |
| SnoU6 | Forward Primer | CATCCGATAA AATTGGAACGA |
|  | Reserves Primer | TTTGTGCGTGTCATCCTTGCG |
